# Chylous Ascites Associated with Advanced Pancreatic Cancer That Improved with Appropriate Treatment: A Case Report

**DOI:** 10.3390/curroncol31030112

**Published:** 2024-03-12

**Authors:** Hiroo Imai, Ken Saijo, Noriko Takenaga, Keigo Komine, Kota Ouchi, Yuki Kasahara, Shiori Ishikawa, Keiju Sasaki, Yuya Yoshida, Hidekazu Shirota, Masanobu Takahashi, Chikashi Ishioka

**Affiliations:** 1 Department of Medical Oncology, Tohoku University Hospital, Sendai 980-8547, Japan; hiroo.imai.d8@tohoku.ac.jp (H.I.); ken.saijo.d6@tohoku.ac.jp (K.S.); keigo.komine.e7@tohoku.ac.jp (K.K.); kota.ouchi.b3@tohoku.ac.jp (K.O.); yuki.kasahara.d8@tohoku.ac.jp (Y.K.); shiori.takahashi.q7@dc.tohoku.ac.jp (S.I.); keiju.sasaki.d6@tohoku.ac.jp (K.S.); yuya.yoshida.c1@tohoku.ac.jp (Y.Y.); hidekazu.shirota.e1@tohoku.ac.jp (H.S.); masanobu.takahashi.a7@tohoku.ac.jp (M.T.); 2 Department of Clinical Oncology, Tohoku University Graduate School of Medicine, Sendai 980-8575, Japan; noriko.takenaga.b3@tohoku.ac.jp

**Keywords:** chylous ascites, octreotide, pancreatic cancer, chemotherapy

## Abstract

Chylous ascites is a rare form of ascites with high triglyceride content arising from the thoracoabdominal lymph nodes in the peritoneal cavity due to various benign or malignant etiologies, including pancreatic cancer. During cancer chemotherapy, the accumulation of ascites can lead to the deterioration of the patient’s general condition, making chemotherapy administration difficult, and resulting in a poor prognosis. We encountered a rare case of chylous ascites complicated by advanced pancreatic cancer. The patient presented with a discrepancy between the shrinkage of the pancreatic cancer and the accumulation of ascites. Therefore, we were able to promptly diagnose chylous ascites by performing biochemical tests. The patient was treated with octreotide, reportedly effective in treating chylous ascites, which rapidly improved the chylous ascites and general condition of the patient, allowing the patient to continue chemotherapy for pancreatic cancer. Therefore, physicians should consider the possibility of chylous ascites when clinically unexplained ascites are observed in patients with advanced cancer. The investigation and treatment of chylous ascites should be initiated as soon as possible.

## 1. Introduction

Chylous ascites is a rare form of ascites with high triglyceride content arising from the thoracoabdominal lymph nodes in the peritoneal cavity due to various benign or malignant etiologies [1]. It occurs in approximately 1 in 20,000 patients and is predominantly caused by surgical complications, trauma, or cirrhosis [2]. A few cases of chylous ascites are associated with solid malignancies, and only three cases associated with pancreatic cancer have been reported [2,3,4].

Here, we encountered a rare case whereby chemotherapy for pancreatic cancer was continued after the appropriate evaluation and treatment of chylous ascites associated with advanced pancreatic cancer.

## 2. Case Presentation

**Figure 1:**
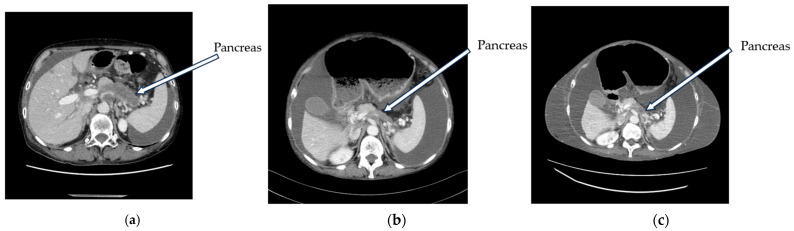
Computed tomography (CT) scan taken during treatment. (**a**) Baseline image prior to initiation of anticancer therapy. Pancreatic cancer, liver metastases, and ascites were shown. (**b**) CT scan taken 56 d after the start of anticancer therapy. While the pancreatic cancer shrunk, the ascites increased. (**c**) CT scan taken at the time of hospitalization. The pancreatic cancer maintained its shrinkage, but the ascites continued to increase and marked subcutaneous edema could be observed.

A 68-year-old woman presented to the hospital with epicardial pain, anorexia, and a weight loss of 7 kg over 3 months. Contrast-enhanced computed tomography (CT) revealed a low-absorptive mass extending from the body of the pancreas to the tail and multiple hepatic nodules. Histological examination using endoscopic ultrasound-guided fine-needle biopsy of the pancreas revealed adenocarcinoma, leading to the diagnosis of pancreatic cancer. CT imaging at the first visit showed pancreatic body–tail cancer, multiple liver metastases, and ascites (Figure 1a). (Tumor markers at this point were CEA 31.5, CA19-9 8374.0). Subsequently, the patient received first-line chemotherapy with gemcitabine and nanoparticle albumin-binding paclitaxel therapy (GnP therapy) at the Department of Medical Oncology, Tohoku University Hospital, Japan. At the initial visit, the patient weighed 64.5 kg, and her Eastern Cooperative Oncology Group (ECOG) performance status (PS) was 0. GnP therapy resulted in the shrinkage of the pancreatic cancer and liver metastases compared to the first CT (Figure 1b) (tumor markers at this point were CEA 7.6, CA19-9 667.0). However, an increase in ascites was observed, resulting in marked anorexia and a worsening of the patient’s ECOG performance status (PS) from 0 to 2. The patient’s body weight further increased to 74.1 kg. An abdominal puncture was performed on an outpatient basis to relieve the subjective symptoms of abdominal distension and to drain the ascites. The collected ascites was milky yellow in color and was subjected to cytological and biochemical analysis. The cytological results were class V, and the patient was found to have peritoneal seeding. Moreover, the ascites contained a high level of triglycerides (222 mg/dL) and was therefore identified as chylous ascites (Table 1).

Subsequently, the patient’s oral intake decreased, her ECOG PS worsened to 4, and she had difficulty living at home. Therefore, she was admitted to the Department of Medical Oncology at Tohoku University Hospital. A third CT scan performed when the patient was admitted to the hospital revealed that the tumor had maintained its shrinkage. However, increased ascites and marked subcutaneous edema were observed (Figure 1c) (tumor markers at this point were CEA 3.6, CA19-9 118.0). As a low-fat, high-protein diet and intravenous nutrition have been reported to be effective in the treatment of chylous ascites [5], a subcutaneous port was inserted and the patient was started on the intravenous high-calorie nutrition. The patient had severe anorexia and was unable to take food orally; therefore, only high-calorie nutrition was administered.

**Figure 2:**
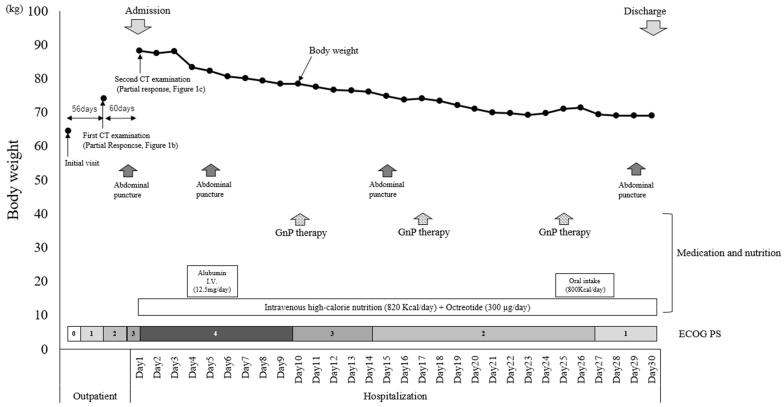
Weight changes during outpatient treatment and hospitalization. Nutrition and medications administered, medical procedures performed, and ECOG PS during the course are also included.

As this patient presented with subileus due to ascites accumulation, octreotide, which is approved for the relief of subjective symptoms of abdominal distension, was also administered (300 µg/day) on the first day after admission. On the fourth day (after starting the octreotide treatment), a decrease in body weight was observed (Figure 2). In addition, the patient’s symptoms of abdominal distension reduced, and the patient was able to walk independently on the 14th day. On the 24th day, oral intake was resumed at the patient’s request and the patient was able to complete an 800 Kcal meal. However, this resulted in weight gain and worsening of the subjective symptoms of abdominal distension. The patient fasted again on the 27th day and was discharged from the hospital on the 30th day (tumor markers at this point were CEA 2.9, CA19-9 84.7), following a policy of continued administration of a mixed infusion of octreotide and intravenous high-calorie nutrition. After discharge, the patient’s general condition remained ECOG PS 1 and she weighed approximately 70 kg. With improvement in her general condition, she complained of hunger and started adequate oral intake at home, but her abdominal distension worsened for a time and her weight increased again. As an exacerbation of biliary ascites was suspected, the patient was instructed to receive high-calorie infusions (mixed with octreotide) and only small amounts of oral intake. With these instructions, the patient remains in good general condition and is receiving GnP therapy for pancreatic cancer on an outpatient basis, which continues to respond.

During hospitalization, abdominal punctures were performed thrice, and ascites was collected. Over time, the milky-white opacity and triglyceride levels in the ascites continued to improve (Table 1).

## 3. Discussion

Chylous ascites is associated with malignancy, cirrhosis, and complications of abdominal surgery in most cases [6]. However, in the present case, the patient had no history or findings suggestive of cirrhosis and no surgical history. Therefore, it is believed that the primary site of pancreatic body–tail cancer and peritonitis metastasis causes the compression of the lymphatic vessels in the abdomen, causing lymphatic fluid to leak into the abdominal cavity. Since 1999, there have only been three case reports of chylous ascites complicated by pancreatic cancer in patients without a history of surgery. Therefore, we believe that this is a rare case.

Chylous ascites is milky [7], with low amylase concentration [8], low bilirubin concentration [9], or a triglyceride concentration >110 mg/dL [10,11,12]. Similarly, in the present case, the biochemical examination of the milky ascites revealed low amylase and bilirubin levels and a high triglyceride level of 222 mg/dL, leading to the diagnosis of cholestatic ascites.

The primary treatment for chylous ascites is dietary adjustment with a low-fat, high-protein diet [13]. However, herein, the patient was unable to take food orally due to abdominal distention at the time of admission. Therefore, it was impossible to treat chylous ascites by adjusting diet. Furthermore, the patient developed subileus, and treatment with octreotide was initiated immediately after admission to relieve the symptoms of abdominal distention. Weight loss began on the fourth day of admission. During hospitalization, the patient lost approximately 15 kg of body weight, the symptoms of abdominal distension improved, and the ECOG PS improved.

Previous case reports reported that treatment with octreotide appears to improve chylous ascites [14,15,16,17]. Octreotide, a somatostatin analog, reduces portal pressure by inhibiting glucagon and other splanchnic intestinal peptides. It also suppresses the exocrine function of the pancreas, resulting in decreased fat absorption from the intestine [6]. Based on this mechanism, octreotide is thought to reduce the incidence of chylous ascites. However, the detailed mechanism underlying the efficacy of octreotide in chylous ascites remains unclear. Moreover, octreotide has not been established as a standard treatment for chylous ascites due to the lack of scientific evidence. However, in our case, octreotide was the only treatment administered after admission. Therefore, the improvement in chylous ascites and the patient’s ECOG PS was attributable to octreotide treatment.

This study had several limitations. First, this was a report of a single case. Second, in this case report, we have not identified the direct cause of the chylous ascites accumulation. A lymphoscintigraphic study with radionuclide is useful in searching for the cause of chylous ascites [18]. However, we have not yet been able to perform this test in this case. It will be performed in the near future. Third, in this case, the cytology of the ascites was class V, and the patient had cancerous peritonitis. Therefore, it cannot be denied that cancerous ascites can be reduced by the treatment efficacy of GnP therapy. However, before the ascites increased, the shrinkage of the pancreatic cancer and liver metastases had already been confirmed by CT. Under these conditions, an increase in the amount of ascites was observed. Malignant ascites usually decreases as the cancer shrinks with cancer chemotherapy [19]. Therefore, given the discrepancy between the shrinkage of the cancer and the progression of ascites accumulation, the ascites cannot be considered to be of cancerous origin. In addition, the triglyceride levels in the ascites, collected three times during hospitalization, decreased over time. Therefore, it is likely that the decrease in ascites was not due to the treatment efficacy of GnP therapy but probably due to the treatment efficacy of octreotide.

In this case, we noticed a discrepancy in the course of the disease: despite the shrinkage of the pancreatic cancer, the patient had an accumulation of ascites. The collected ascites was then submitted to biochemical examinations, and the ascites was diagnosed as chylous ascites. Since ascites accumulation is often complicated by intestinal obstruction, it is acceptable to administer octreotide, which is approved for the reduction in abdominal distention due to intestinal obstruction. In the case of the unexplained accumulation of ascites during anticancer therapy, the attending physician should collect the ascites and perform biochemical examinations. In case of a diagnosis of chylous ascites, octreotide treatment may reduce ascites, maintain the patient’s general condition, allow continuation of anticancer therapy, and improve the patient’s prognosis.

## 4. Conclusions

Herein, we report a rare case of chylous ascites associated with advanced pancreatic cancer managed by octreotide and dietary adjustments.

## Figures and Tables

**Table 1:** Results of biochemical and cytological examination of ascites.

	Outpatient	Hospitalization		
		Day 5	Day 15	Day 29
Glucose (mg/dL)	133	147	150	152
Total protein (g/dL)	2.1	1.8	2.2	2.7
LDH (U/L)	92	82	95	99
Amylase (IU/L)	18	21	22	27
CEA (ng/nL)	3.9	4.5	5.4	4.3
Total bilirubin (mg/dL)	0.2	0.2	0.3	0.2
Triglyceride (mg/dL)	222	181	57	35
Cytology	Class V			

LDH: lactate dehydrogenase, CEA: carcinoembryonic antigen.

## Data Availability

The data used to support the findings are presented in the tables and figures included in the manuscript. The detailed retrospective observational data used to support the findings of this study are available from the first author (Hiroo Imai; e-mail: hiroo.imai.d8@tohoku.ac.jp) upon reasonable request. None of the data in the current study contained personal identifiers and were kept confidential. The data are not publicly available for ethical reasons. Further inquiries can be directed to the corresponding authors.

## References

[1] Kim J., Won J.H. (2016). Percutaneous Treatment of Chylous Ascites. Tech. Vasc. Interv. Radiol..

[2] Satala C.B., Bara T.J., Jung I., Tudorache V., Gurzu S. (2021). Chylous Ascites, Unusual Association with Ductal Pancreatic Adenocarcinoma with Plasmacytoid Morphology: A Case Report and Literature Review. Surg. J..

[3] Wagayama H., Tanaka T., Shimomura M., Ogura K., Shiraki K. (2002). Pancreatic cancer with chylous ascites demonstrated by lymphoscintigraphy: Successful treatment with peritoneovenous shunting. Dig. Dis. Sci..

[4] Fangsaard P., Puriwekin J., Phattraprayoon N., Ungtrakul T. (2022). Unusual Presentation of Bilateral Chylothorax and Chylous Ascites with Pancreatic Adenocarcinoma: A Case Report. Case Rep. Oncol..

[5] Duletzke N.T., Kiraly L.N., Martindale R.G. (2023). Chylothorax and chylous ascites: Overview, management, and nutrition. Nutr. Clin. Pract. Off. Publ. Am. Soc. Parenter. Enter. Nutr..

[6] Bhardwaj R., Vaziri H., Gautam A., Ballesteros E., Karimeddini D., Wu G.Y. (2018). Chylous Ascites: A Review of Pathogenesis, Diagnosis and Treatment. J. Clin. Transl. Hepatol..

[7] Tulunay G., Ureyen I., Turan T., Karalok A., Kavak D., Ozgul N., Ocalan R., Tapisiz O.L., Boran N., Kose M.F. (2012). Chylous ascites: Analysis of 24 patients. Gynecol. Oncol..

[8] Assumpcao L., Cameron J.L., Wolfgang C.L., Edil B., Choti M.A., Herman J.M., Geschwind J.F., Hong K., Georgiades C., Schulick R.D. (2008). Incidence and management of chyle leaks following pancreatic resection: A high volume single-center institutional experience. J. Gastrointest. Surg. Off. J. Soc. Surg. Aliment. Tract.

[9] Malik H.Z., Crozier J., Murray L., Carter R. (2007). Chyle leakage and early enteral feeding following pancreatico-duodenectomy: Management options. Dig. Surg..

[10] Kuboki S., Shimizu H., Yoshidome H., Ohtsuka M., Kato A., Yoshitomi H., Furukawa K., Miyazaki M. (2013). Chylous ascites after hepatopancreatobiliary surgery. Br. J. Surg..

[11] Baek S.J., Kim S.H., Kwak J.M., Kim J. (2013). Incidence and risk factors of chylous ascites after colorectal cancer surgery. Am. J. Surg..

[12] Aoki H., Takakura N., Shiozaki S., Matsukawa H. (2010). Milk-based test as a preventive method for chylous ascites following pancreatic resection. Dig. Surg..

[13] Lizaola B., Bonder A., Trivedi H.D., Tapper E.B., Cardenas A. (2017). Review article: The diagnostic approach and current management of chylous ascites. Aliment. Pharmacol. Ther..

[14] Lee I.H., Kim S.G., Park K.S., Ahn D.J., Kim M.K. (2023). Chylothorax associated with primary membranous nephropathy: A case report. Ann. Palliat. Med..

[15] Rashid R., Shafi Ahmed S., Mahmud S. (2022). Congenital Chylous Ascites: A Rare Cause of Infantile Ascites Treated with MCT-Based Diet and Octreotide. JPGN Rep..

[16] Singh H., Pandit N., Krishnamurthy G., Gupta R., Verma G.R., Singh R. (2019). Management of chylous ascites following pancreaticobiliary surgery. JGH Open Open Access J. Gastroenterol. Hepatol..

[17] Bhatia C., Pratap U., Slavik Z. (2001). Octreotide therapy: A new horizon in treatment of iatrogenic chyloperitoneum. Arch. Dis. Child..

[18] Tai E., Min A., Rajan D.K. (2021). A Single-Center Experience with Percutaneous Interventional Management of Refractory Chylous Ascites. Can. Assoc. Radiol. J. J. L’association Can. Des Radiol..

[19] Yamao T., Shimada Y., Shirao K., Ohtsu A., Ikeda N., Hyodo I., Saito H., Iwase H., Tsuji Y., Tamura T. (2004). Phase II study of sequential methotrexate and 5-fluorouracil chemotherapy against peritoneally disseminated gastric cancer with malignant ascites: A report from the Gastrointestinal Oncology Study Group of the Japan Clinical Oncology Group, JCOG 9603 Trial. Jpn. J. Clin. Oncol..

